# Are disadvantaged children more likely to be excluded from analysis when applying global positioning systems inclusion criteria?

**DOI:** 10.1186/s13104-018-3681-2

**Published:** 2018-08-13

**Authors:** Suzanne Mavoa, Karen Lamb, David O’Sullivan, Karen Witten, Melody Smith

**Affiliations:** 1 Non Communicable Disease Unit, Melbourne School of Population & Global Health, The University of Melbourne, Level 5, 333 Exhibition Street, Melbourne, VIC 3000 Australia; 2 SHORE and Whariki Research Centre, School of Public Health, Massey University, Auckland, New Zealand; 3 Institute for Physical Activity and Nutrition (IPAN), School of Exercise and Nutrition Sciences, Deakin University, Geelong, Australia; 4 Department of Geography, University of California, Berkeley, 505 McCone Hall, Berkeley, 94720-4740 USA; 5 School of Nursing, The University of Auckland, Private Bag 92019, Auckland, 1142 New Zealand

**Keywords:** GPS, Inclusion criteria, Missing data, Bias, Exposure, Children, Equity

## Abstract

**Objective:**

When using global positioning systems (GPS) to assess an individual’s exposure to their environment, a first step in data cleaning is to establish minimum GPS ‘inclusion criteria’ (a set of rules used to determine which GPS data are able to be included in analyses). Care is needed at this stage to avoid any data exclusion (data loss) systematically biasing results in terms of characteristics of the environment and participants. The extent of potential systematic bias in sample retention due to GPS data loss and application of GPS inclusion criteria is unknown. The aim of this study was to describe differences in sample size and socio-demographic characteristics of the retained sample when applying three different GPS inclusion criteria. The study assessed 7-day GPS data collected from children (aged 9–13 years) recruited from nine schools in Auckland, New Zealand as part of the Kids in the City study.

**Results:**

Participants from ethnic minorities and those attending schools in lower socioeconomic areas were disproportionately excluded from the retained samples. This highlights potential equity implications in basing the assessment of exposure—which ultimately influences research results on the relationship between environment and health—on non-representative GPS data.

## Introduction

Increasingly, researchers are using global positioning systems (GPS) to track where people go, and to more precisely assess exposure to the environment compared to self-report or the residential neighbourhood [1]. Researchers have used GPS to explore relationships between the environment and diverse outcomes such as diet [2] unhealthy food purchasing [3], physical activity [4], and alcohol use [5]. GPS has also been used to assess exposure to pollution [6], routes travelled [7], independent mobility [8], and time spent indoors or outdoors [9].

Missing and erroneous data is a known issue with GPS [10–12]. GPS data may not be recorded for a number of reasons including signal drop out due to loss of satellite visibility, signal acquisition times, dead batteries, or data loss during download [12–16]. Recorded GPS data may be erroneous due to participants not wearing/losing the GPS device and signal scatter due to loss in satellite visibility [12–16]. Some data loss could be associated with participant characteristics and lead to systematic bias in study results [17].

Similarly, there is potential systematic bias due to application of GPS inclusion criteria used to determine whether a participant has sufficient data to reliably estimate behaviours of interest. Despite this, few GPS studies report their GPS inclusion criteria, and there are no standards among those that do [17].

Application of inclusion criteria has resulted in significant differences in characteristics of samples retained compared to those excluded for analysis of data from other wearable devices such as accelerometers [18–20]. However, no research has investigated the impact of applying inclusion criteria to GPS data. Furthermore, Meseck et al. [11] is the only study that has evaluated bias associated with GPS data loss. Therefore, this study aims to compare descriptive differences in sample size and sociodemographic characteristics of excluded/included participants when applying three different GPS inclusion criteria.

## Main text

### Methods

Data from the Kids in the City (KITC) study were used. Detailed methods are described elsewhere [21]. Children aged 8–13 years (109 males, 141 females) from Auckland, New Zealand were recruited from nine schools with diverse built environment characteristics and school socio-economic status (SES).

Participant demographic characteristics (sex, age, ethnicity, number of household cars) were collected from parents/caregivers in a computer-aided telephone interview. Number of household cars was a proxy for household SES. School SES data was sourced from the New Zealand Ministry of Education.

The shortest road network distance between each participant’s home and the nearest school entrance was calculated using geographic information systems (GIS). Home addresses were geocoded and school entrance points manually digitised based on entrance locations visible in satellite imagery. A 2011 ‘improved road centreline’ dataset was downloaded from http://www.koordinates.com. Non-walkable road segments (motorways and on-ramps) were removed before analysis. GIS analyses were undertaken in ArcGIS 9.3 (ESRI Inc, Redlands, CA).

Seven consecutive days of GPS data were collected using QStarz BT-Q1000 and BT-Q1000XT units (Qstarz International Inc., Taiwan). The only relevant difference between the units was the greater storage capacity of the BT-QT1000XTs. Both units had sufficient storage for the study.

Data were collected during school terms in 2011 and 2012. GPS units were worn on a belt and collected data every 10 s. Participants recorded when they put on and took off the belt. During weekdays the research team visited the school to download the previous day’s GPS data and charge units. On Fridays the children were given chargers and instructed to charge the units each weekend night. Weekend GPS data were downloaded by the research team on Monday at school.

Three GPS inclusion criteria were developed, applied and assessed.

#### Inclusion criterion 1

Inclusion criterion 1 was as inclusive as possible while also requiring minimally valid GPS and address data.

1. The home address was able to be geocoded; and
2. Participants reported a single home address; and
3. GPS data were recorded at the home address; and
4. Three or more hours of GPS data were collected during the 7-day data collection period.

#### Inclusion criterion 2

Investigating spatio-temporal location patterns from GPS data requires sufficient data points on different days of the week and times of the day. Ideally, this would mean using an inclusion criterion with a high minimum number of hours per day for different days of the week. However, participants with missing data may also have periods of high quality GPS data (e.g., due to spending time in locations with poor satellite visibility) and strict inclusion criteria may exclude otherwise potentially useful data. Therefore, the following approach was taken.

First, the GPS data were divided into three categories: weekdays before school, weekdays after school, and weekends. Weekdays before school included GPS points recorded on weekdays, starting from the time the GPS was put on and ending at the start of school (based on school start time). Weekdays after school included GPS points recorded on weekdays from the end of school (based on school end time) and ending at the time the GPS was removed for the day. The different school start and end times were taken into account when categorising the GPS data. Weekends included all GPS data recorded on a Saturday or Sunday.

Next, the following additional inclusion criteria were applied to the complete GPS dataset:

1. At least 2 weekdays with at least 30 min before school data; and
2. At least 2 weekdays with at least 2 h after school data; and
3. At least 5 h of total weekend data.

The number of valid days and the duration of valid GPS data were determined by considering the population, the purpose of the broader KITC study, and building on criteria used in published literature [22, 23].

#### Inclusion criterion 3

The third criterion was based on inclusion criteria that had been applied to accelerometer data in the KITC study [24]:

1. Weekdays required at least three non-school hours of GPS data and weekends required at least 7 h of GPS data; and
2. Each participant required at least two valid days of weekday data and one valid weekend day.

The number and percentage of included participants within categories of important demographic characteristics (school, sex, age, ethnicity, number of cars, distance to school) were calculated for each sample (full, criterion 1, criterion 2, criterion 3). Percentage retention for each category (e.g., number of males in criterion 3/number of males in full sample), and the percentage in each category compared to the total participants in the criterion (e.g., number of males in criterion 3/number of participants in criterion 3) were calculated for each criteria. For each characteristic, Pearson Chi square tests were used to compare the proportions between the full sample and each of the criterion.

### Results

One participant did not supply any demographic or GPS data, leaving 253 participants included in this analysis.

Table 1 presents characteristics of the full sample alongside those for the sample under each of the three GPS criteria. Increasingly strict inclusion criteria reduced sample size (up to 81% loss for criteria 3).

**Table 1:** Characteristics of the three GPS datasets arising from application of the three inclusion criteria

	Full sample n (% of full sample)	Criterion 1 n (% of criterion 1 sample)	p-value^a^	Criterion 1 (% of full sample)	Criterion 2 n (% of criterion 2 sample)	p-value^a^	Criterion 2 (% of full sample)	Criterion 3 n (% of criterion 3 sample)	p-value^a^	Criterion 3 (% of full sample)
Total	253 (100.0)	236 (100.0)		93.3	85 (100.0)		33.6	48 (100.0)		19.0
School (SES)			0.345			0.008			0.000	
1 (low)	30 (11.9)	25 (10.6)		83.3	3 (3.5)		10.0	3 (6.3)		10.0
2 (low)	23 (9.1)	22 (9.3)		95.7	4 (4.7)		17.4	0 (0)		0.0
3 (low)	26 (10.3)	23 (9.7)		88.5	7 (8.2)		26.9	3 (6.3)		11.5
4 (low)	26 (10.3)	25 (10.6)		96.2	6 (7.1)		23.1	1 (2.1)		3.8
5 (medium)	30 (11.9)	29 (12.3)		96.7	12 (14.1)		40.0	8 (16.7)		26.7
6 (medium)	25 (9.7)	23 (9.7)		92.0	13 (15.3)		52.0	4 (8.3)		16.0
7 (high)	28 (11.1)	28 (11.9)		100.0	11 (12.9)		39.3	5 (10.4)		17.9
8 (high)	13 (5.1)	12 (5.1)		92.3	5 (5.9)		38.5	4 (8.3)		30.8
9 (high)	52 (20.6)	49 (20.8)		94.2	24 (28.2)		46.2	20 (41.7)		38.5
Sex			0.843			0.991			0.562	
Female	143 (56.5)	133 (56.4)		93.0	48 (56.5)		33.6	27 (56.3)		18.9
Male	110 (43.5)	103 (43.6)		93.6	37 (43.5)		33.6	21 (43.8)		19.1
Age (years)			0.799			0.068			0.034	
8	4 (1.6)	4 (1.7)		100.0	1 (1.2)		25.0	1 (2.1)		25.0
9	69 (27.3)	64 (27.1)		92.7	25 (29.4)		36.2	17 (35.4)		24.6
10	153 (60.5)	142 (60.2)		92.8	45 (52.9)		29.4	23 (47.9)		15.0
11	21 (8.3)	20 (8.5)		95.2	12 (14.1)		57.1	5 (10.4)		23.8
12	4 (1.6)	4 (1.7)		100.0	2 (2.4)		50.0	2 (4.2)		50.0
13	2 (0.8)	2 (0.8)		100.0	0 (0)		0.0	0 (0)		0.0
Ethnicity			0.006			0.008			0.006	
European	57 (22.5)	54 (22.9)		94.7	26 (30.6)		45.6	20 (41.7)		35.1
Indian/Asian/other	67 (26.5)	65 (27.5)		97.0	30 (35.3)		44.8	16 (33.3)		23.9
Māori	31 (12.3)	28 (11.9)		90.3	4 (4.7)		12.9	0 (0)		0.0
Not stated	15 (5.9)	13 (5.5)		86.7	5 (5.9)		33.3	3 (6.3)		20.0
Other Pacific Islander	45 (17.8)	42 (17.8)		93.3	12 (14.1)		26.7	4 (8.3)		8.9
Samoan	38 (15.0)	34 (14.4)		89.5	8 (9.4)		21.1	5 (10.4)		13.2
Number of cars			0.829			0.677			0.873	
0	24 (9.5)	23 (9.7)		95.8	5 (5.9)		20.8	2 (4.2)		8.3
1	106 (41.9)	100 (42.4)		94.3	39 (45.9)		36.8	20 (41.7)		18.9
2	79 (31.2)	73 (30.9)		92.4	26 (30.6)		32.9	19 (39.6)		24.1
≥ 3	28 (11.1)	26 (11.0)		92.9	10 (11.8)		35.7	4 (8.3)		14.3
Not stated	16 (6.3)	14 (5.9)		87.5	5 (5.9)		31.3	3 (6.3)		18.8
Distance to school (m)			0.113			0.117			0.055	
0–400	44 (17.4)	40 (16.9)		90.9	10 (11.8)		22.7	5 (10.4)		11.4
401–800	69 (27.3)	65 (27.5)		94.2	27 (31.8)		39.1	16 (33.3)		23.2
801 –1200	50 (19.8)	48 (20.3)		96.0	11 (12.9)		22.0	5 (10.4)		10.0
1201–2000	35 (13.8)	34 (14.4)		97.1	13 (15.3)		37.1	6 (12.5)		17.1
2001–10,000	41 (16.2)	39 (16.5)		95.1	18 (21.2)		43.9	10 (20.8)		24.4
> 10,000	12 (4.7)	9 (3.8)		75.0	5 (5.9)		41.7	5 (10.4)		41.7
Not stated	2 (0.8)	1 (0.4)		50.0	1 (1.2)		50.0	1 (2.1)		50.0

^a^p-values from Chi square tests comparing included versus excluded participants for each criterion

With the exception of sex, percentage of the sample retained at criteria 1–3 varied for the socio-demographic characteristics assessed. Different distance to school and age categories had similar percentage retentions at criterion 1, but by criterion 2 and 3 varied more. There was no clear pattern between distance to school and percentage retained, nor between age and percentage retained.

The most marked variation in percentage retained was for school attended and ethnicity. Only 95.7, 17.4, and 0% of participants in school 2 were retained in criteria 1, 2, and 3 respectively compared with 96.7%, 40.0%, 26.7% of participants from school 6. None of the Māori participants and a relatively low percentage of Samoan (13.2%) and Other Pacific Island (9.1%) participants were retained when applying criterion 3, compared to 35.1% of Europeans and 23.9% of Indian/Asian/other.

Table 1 also presents percentage of participants retained in each socio-demographic category in relation to the total number of participants in each criterion, revealing how the loss of numbers in the sample affects the representation in the sample. There was little change in the representation of females/males when each criterion was applied. However, the same could not be said for the other socio-demographic characteristics, with the most notable differences occurring again for school attended and ethnicity.

Table 1 also presents p-values from the Chi square tests to provide an estimate of bias. There was evidence of a difference in proportions between the full sample and at least one of the criterion within ethnicity (criterion 1, 2, 3), school (criterion 2, 3) and age (criterion 3) categories.

### Discussion

This study aimed to describe the impact of applying different GPS inclusion criteria. While it is obvious that the application of increasingly strict inclusion criteria will reduce the sample size, this study highlighted the dramatic reduction in sample size in our GPS dataset of New Zealand children. Of greater concern was the finding that the sample retention exhibited sociodemographic bias, and likely environmental bias due to the location of schools in diverse environments. Yet inclusion criteria are important to ensure data are as representative of participants’ behaviour as possible. Ultimately, there is a trade-off between ideal criteria and maximising the retained sample size. Improving compliance of different subgroups, more comprehensive analysis of this trade-off, and the development of standardised GPS inclusion criteria are important knowledge gaps for researchers to address in future research.

As demonstrated here, applying certain inclusion criteria can result in small sample sizes, emphasising the importance in taking care to minimise data loss. Bias due to data loss may occur due to participant and device factors, some of which may be reduced by researchers following strategies such as testing GPS devices prior to use [12], setting up the devices to only collect necessary data (and save memory) with appropriate epochs [12], using devices that don’t require participants to charge them, checking the device is working during data collection [21], providing participants with clear instructions [12], sending reminder messages to participants to charge the device [12, 18], and providing a voucher as an incentive to participants [18].

Results highlighted differences in sample retention between schools. When applying criterion 3 the percentages of participants retained from schools 1, 2, 3, and 4 were lower than the other five schools.

Our descriptive results demonstrated striking differences in retention of participants by ethnicity, adding impetus to addressing a widely acknowledged challenge within child health research: that of engaging children and families from lower socioeconomic backgrounds and minority ethnic populations [25–27]. Māori and Pacific Island participants at schools with lower socio-economic status were disproportionately excluded when applying stricter inclusion criteria. Māori and Pacific Islanders and those with lower socio-economic status, also have poorer health [28, 29], highlighting potential equity implications in basing the assessment of exposure—which impacts research results—on non-representative GPS data.

### Conclusion

GPS allows researchers to measure exposure to the environment more precisely than self-report or using the residential neighbourhood as a proxy for exposure. In doing so, it is important to ensure that the GPS data represent the population and behaviours of interest. Researchers using GPS data should consider and report application of GPS inclusion criteria where relevant. In deciding on appropriate inclusion criteria, it is important to consider the research question and use of GPS data. Appropriate criteria may vary for different research questions and study populations. Assessment of socioeconomic and environmental biases in missing GPS data is needed to ensure appropriate interpretation of results.

## Limitations

There may have been bias in the selection of participants into the study, which we were unable to account for. While our findings are sample specific, they highlight a potential issue that future studies could test by collecting and analysing and reporting details of GPS data loss.

This study did not assess environmental attributes. However, since the schools were located in different environments, it is likely that there would have been an environmental bias in the retained samples.

GPS data were only collected for 7 consecutive days, which are arguably not representative of typical behaviour. However, GPS data quality reduces with longer measurement periods [13].

## Abbreviations

GIS: geographic information systems
GPS: global positioning system
KITC: Kids in the City
SES: socio-economic status
